# In vitro safety of power injection of contrast media through central venous hemodialysis catheters

**DOI:** 10.1177/11297298251333014

**Published:** 2025-04-14

**Authors:** Nicholas A White, Aart J van der Molen, Ronald W A L Limpens, Jacinta J Maas, Koen E A van der Bogt, Tim Horeman, Joris I Rotmans

**Affiliations:** 1 Department of BioMechanical Engineering, Delft University of Technology, Delft, The Netherlands; 2 Department of Internal Medicine, Leiden University Medical Centre, Leiden, The Netherlands; 3 Department of Radiology, Leiden University Medical Centre, Leiden, The Netherlands; 4 Department of Cell and Chemical Biology, Leiden University Medical Center, Leiden, The Netherlands; 5 Department of Intensive Care, Leiden University Medical Center, Leiden, The Netherlands; 6 Department of Surgery, Leiden University Medical Center, Leiden, The Netherlands; 7 University Vascular Centre West, Leiden | The Hague | Delft, The Netherlands

**Keywords:** Contrast media, power injection, central venous catheters, hemodialysis, electron microscopy

## Abstract

**Objective:**

Central venous catheters (CVCs) provide direct access to the central circulatory system, commonly used in hemodialysis and intensive care units for drug administration. Although uncertified for the procedure, CVCs are sometimes used for power injection of contrast medium (CM) during CT scans to avoid peripheral intravenous catheter placement. Previous studies suggest this practice is safe, but incidents are reported. This study aims to measure intraluminal pressure during CM injection through CVCs and assess its impact on the luminal surface to guide responsible clinical use.

**Materials and methods:**

An experimental in vitro test setup was developed. Four samples each of three different types of unused CVCs were used. Strain gauges were applied to the exterior walls of either the inflow or outflow lumen of the CVC. These gauges measured material deformation due to intraluminal pressure during CM injections at rates of 4.5 and 8 mL/s, each performed five times. Strain data were calibrated against known pressures in a static system. Three CVCs of each type were then pressurized until bursting, and one was subjected to microscopic analysis of the luminal surfaces.

**Results:**

Intraluminal pressures measured (97–545 kPa or 14–79 PSI) were below the burst pressure (779–1248 kPa or 113–181 PSI) in all instances. Strain regression analysis shows a statistically significant (*p* < 0.01) trend over 10 injections in all CVCs tested except one, indicating material fatigue. Surface microscopy revealed surface micro-cracks from repeated injections, suggesting material damage.

**Conclusions:**

The intraluminal pressures from power injections of CM are sufficiently low to prevent CVC bursting. While incidental use for CM injection appears safe, repeated use may cause material damage.

## Introduction

Hemodialysis patients frequently utilize a central venous catheter (CVC) as vascular access to transport blood to and from the dialysis machine. Similar CVCs may also be used for administration of drugs to patients in intensive care units. For diagnostic purposes, these patients may need CT scans with radiopaque contrast medium (CM). Bolus intravenous injection of CM using a power (pressure) injector is the preferred method for CM administration for CT examinations of the neck, chest, and abdomen.^1,2^ This is usually achieved with an intravenous needle in the forearm, with typical CM flow velocities around 3–6 mL/s.^3–5^ However, hemodialysis patients often have poor peripheral veins, necessitating preservation for future arteriovenous access surgery. Studies have indicated safe in vitro pressure levels for CM injection through various types of CVCs,^6–12^ and some clinical evidence of safe use.^13–15^ Concerns remain about catheter-related complications due to high pressures in these devices that are often not certified for this usage, with several incidents of rupture and tip displacement reported.^16–18^ Clinical guidelines remain limited.^19^

Clinically, power injector pressures are capped at 2000–2250 kPa (300–325 PSI), but it cannot be claimed that the pressure is constant throughout a dynamic, flowing system. There must be a pressure gradient for fluids to flow. The pressure gradient depends on many variables, including viscosity, flow velocity, friction of the tube surface, and geometry. Although the pressure displayed on the power injector thus overestimates the pressure downstream (e.g. in a CVC), the non-uniform geometry of CVCs could cause local increases in pressure and wall stress.^20^ It is crucial that pressure is measured at the correct location to obtain the most accurate data on intraluminal pressure in CVCs during CM injection to properly assess safety.

Moreover, pressure transducers in a flow circuit interfere with the flow, often requiring a three-way connection that introduces static fluid dead space. This dead space can impede accurate pressure measurement due to its lack of direct pressure response to fluid flow and the flow distortions it introduces. In systems with rapidly changing flow rates, dead space can dampen fluid oscillations and distort pressure readings, contributing to significant pressure measurement differences in previous studies (14–483 kPa (2–70 PSI) at 4.5 mL/s with similar catheters).^6,7,11,12^ As a non-invasive method to measure pressure without the intrinsic drawbacks of pressure transducers, strain gauges may pose a solution.

The present study aims to equip healthcare professionals with the knowledge required to make informed decisions regarding the usage of commonly used CVCs for power injection of CM by presenting a comprehensive study of intraluminal pressure during injection, material fatigue, bursting pressures, and surface analysis.

## Materials and methods

### Central venous catheters

**Figure 1.**
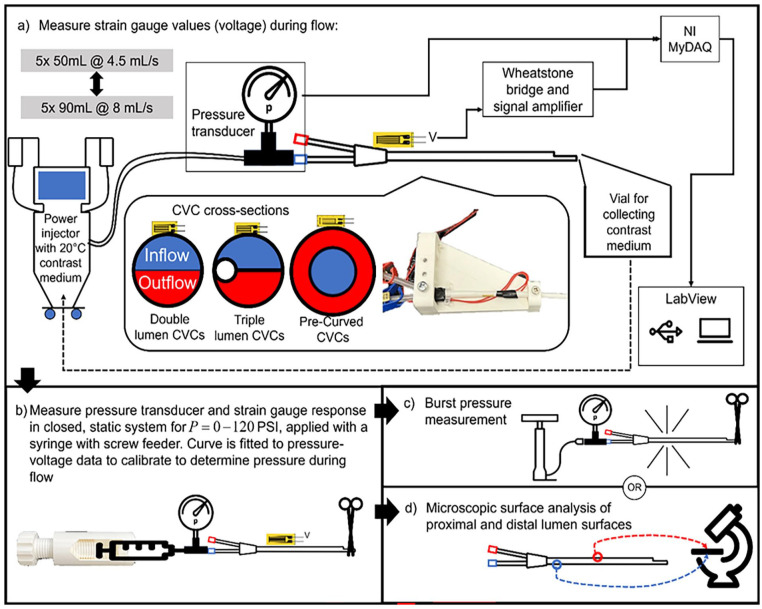
The experimental setup for testing the central venous catheters (CVCs): (a) the catheters with strain gauges are clamped and connected to a contrast medium injector with a pressure transducer placed at the inlet of the catheter. CVC cross-sections are also shown. Blue indicates the inflow (flow to the body) lumen, and red indicates outflow (flow from the body). (b) The strain gauges are then calibrated through static pressure. The pressure transducer values are stored together with the strain gauge voltages. Next the pressure transducer and strain gauge voltages are processed, and a curve fit is generated. Intraluminal pressure during injection is determined through this curve fit. (c) Catheters are finally pressurized until burst; or (d) microscopic surface analysis is performed on the lumina of 1 sample of each catheter types tested.

Each CVC type has an inflow lumen, used for transport of fluid to the body, and an outflow lumen, for fluid transported out of the body. Figure 1(a) shows the differences in cross-sections. Four samples each of three different types of new, unused CVCs were collected and tested:

- 13 F, 250 mm GamCath Dolphin Protect High Flow Double Lumen straight catheter (Baxter, Deerfield, IL, USA)
- 13 F, 250 mm GamCath High Flow Triple Lumen straight catheter (Baxter, Deerfield, IL, USA)
- 15.5 F, 200 mm Jet Medical Short-Term Free Flow pre-curved catheter (Jet Medical SA, La Chaux-de-Fonds, Switzerland)

### Experimental setup

The CVCs are tested by repeated injection of CM and recording the response with strain gauges. The strain gauges are then calibrated with a pressure transducer in a closed system with static pressure in which pressure is equal everywhere and in all directions. Calibration is performed after the CM injection; the pressure necessary to properly calibrate the sensors must exceed the pressure induced by the CM, and therefore may damage the CVCs and distort the pressure recordings if performed prior to the power injections. Finally, the CVCs are either burst or dissected for microscopic surface analysis. The experimental setup is displayed in Figure 1.

**Figure 2.**
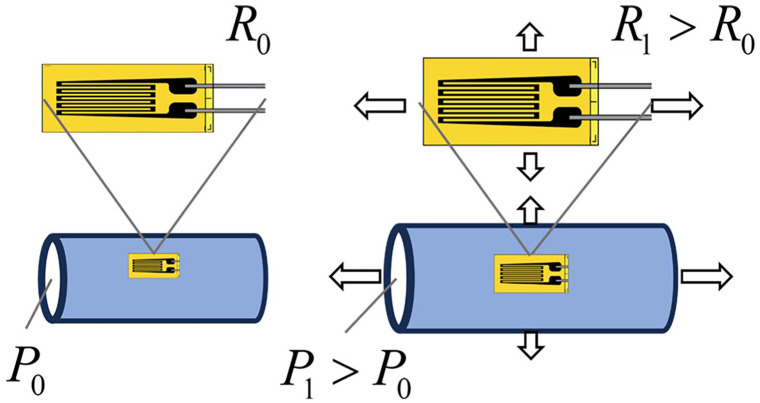
Working principle of a strain gauge. The strain gauge is placed on a surface and the resistance *R* is continuously measured, of which the value is *R*_0_ at rest with intraluminal pressure *P*_0_. When the material stretches, for example, due to a higher intraluminal pressure *P*_1_, the strain deforms with the material. The resistance of the deformed gauge *R*_1_ will increase due to the increase in length and decrease in cross-sectional diameter of the wire. This can then be measured as a voltage change when amplified in a Wheatstone bridge.

The working principle of a strain gauge is shown in Figure 2. Deformation of the material on to which the gauges are placed results in deformation of the thin wires. This in turn results in a change in resistance which can be accurately measured. Materials deform predictably when stress is applied. Intraluminal pressure introduces such stresses in the wall of the CVCs, so strain gauges may be utilized to measure this deformation. The strain can thus be related to an intraluminal pressure when calibrated with a pressure transducer in a static closed system. Additionally, such strain gauges are typically used to determine material wear and fatigue over time. As such, they may provide information relating to such effects occurring in the CVC material with CM injection.

A GFLAB-3-70 Low Elastic Strain Gauge (Tokyo Measuring Instruments Laboratory Co., Tokyo, Japan) was fixed on the inlet side of each CVC. This position was chosen because it is where the pressure is expected to be highest. Placing the strain gauge on the inflow lumen (blue, shown in Figure 1(a)) was preferred as this lumen is typically used for administration of fluids. Standard strain gauge cyanoacrylate adhesive (Tokyo Measuring Instruments Laboratory Co., Tokyo, Japan) was used. The strain gauge was placed in a Wheatstone bridge to amplify the voltage change and correct the signal for effects such as temperature change. To achieve temperature compensation, the other gauges in the bridge were placed on an additional, unused CVC made of the same material. Application of pressure to the CVC, caused a resistance change in the strain gauge, resulting in a change in voltage over the bridge. This voltage signal was further amplified by a CPJ Strain Gauge Conditioner (Scaime, Juvigny, France) and read in LabVIEW through a NI MyDAQ (National Instruments, Austin, TX, USA). Each tested CVC sample was fixated at two points (Figure 1(a)) to ensure no deformation of the catheter occurs, other than induced by pressure.

The inflow lumen of each CVC was connected to a FlowSens^®^ syringeless soft bag CM injector (Guerbet, Paris, France) with standard consumable tubing and with a PU5405 pressure transducer (ifm GmbH, Essen, Germany) fastened with a three-way stopcock just proximal to the CVC connector. The system was flushed with saline and primed so that there was CM iobitridol 350 mgI/mL (Xenetix, Guerbet, Paris, France) in the CVC prior to starting measurements. The test setup and all fluids used were operated at room temperature as strain gauges are sensitive to temperature changes. Since viscosity decreases as temperature rises, higher pressures are required at lower temperatures. This represents a worst-case scenario where the fluid is administered without prior heating.^21^ The CM exiting the tip of the CVC was collected in a vial and reused.

### Flow measurement

First, 50 mL of CM was injected into each CVC at 4.5 mL/s over 11 s,^6,7,11,12^ and both the pressure transducer and strain gauge values were recorded. Due to the viscoelasticity (i.e. non-direct strain response) of the material, a 5 min resting time is needed prior to commencing the next measurement. This process was repeated five times. Next, 90 mL was injected at 8 mL/s over 11 s, corresponding to the protocol with the highest flow rate used in our center. The same 5-min resting time was applied, for five measurements. The maximum pressure in the power injector was also recorded. For half of the samples, the order was reversed, with 90 mL injections performed first, followed by 50 mL injections. Finally, the CVCs were flushed with saline.

#### Pre-curved CVCs

The pre-curved CVCs do not have the same “double-D” cross section, but an inner (inflow) and outer (outflow) lumen (Figure 1(a)). Due to this geometry it was not possible to directly attach a strain gauge to the wall of the preferred inflow lumen. Instead, the strain gauges were secured to the wall of the outflow (outer, red) lumen, and measurements were taken through this lumen. However, this lumen is not typically used for administration of fluids. Therefore injection with the power injector was repeated with both lumina and maximum injection pressure was recorded for both lumina. This provides an indication of pressure differences between these lumina. The measurement of the inner lumen was performed in three of the four samples. The inner lumen in the remaining sample was spared for microscopic surface analysis.

### Sensor calibration and data processing

After completing the flow measurements, the tip of each catheter was clamped and the power injector was disconnected. A syringe filled with room temperature water and a screw feeder was connected to the three-way stopcock to create a closed and static system. The strain gauge was calibrated by gradually increasing the pressure to ~800 kPa (~120 PSI) over 2 min three times per CVC, and recording the strain gauge voltage together with transducer values. This data was processed in MATLAB (MathWorks, Natick, Massachusetts), in which the calibration data was zero-shifted, and a third-order polynomial was fitted. The maximum intraluminal pressure of each measurement was determined by inserting the zero-shifted maximum gauge value into the curve fit of the respective CVC. To assess material fatigue (permanent strain) and damage of the CVC material after CM injection, the resting voltages were recorded of the strain gauge after the 5-min relaxation periods. Linear regression was applied to these values to assess for a non-zero linear coefficient with 95% confidence.

### Burst pressure test

Three samples each of the three different CVC types were then connected to a high-pressure manual bicycle pump with a calibrated Gems 3300 1600 kPa pressure transducer (Gems, Plainville, CT, USA) fastened through a three-way stopcock just proximal to the CVC connector. The catheter was filled with room temperature water and the tip was sealed with a medical clamp. Pressure was increased until burst.

### Microscopic analysis

The remaining CVCs were dissected. Surface samples from the inlet ends of the lumina subjected to power injection of CM were collected and sectioned. The samples from the unused, outflow lumina of the same CVCs were also taken as control. The samples were mounted on Scanning Electron Microscopy stubs with double sides carbon stickers. Before imaging, all samples were sputter-coated with a layer of Gold/Palladium. Images were recorded in a GeminiSEM 300 Scanning Electron Microscope (Zeiss, Oberkochen Germany), operated at 5 kV.

## Results

### Calibration

**Figure 3.**
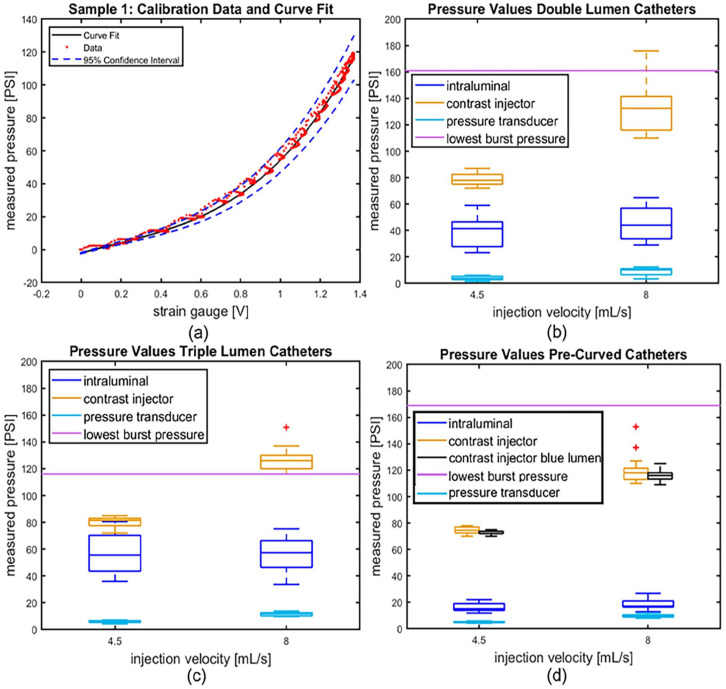
(a) The calibration data, fitted third-order polynomial and its 95% confidence bounds of catheter sample 1, used to determine intraluminal pressure with the strain gauge. (b–d) Boxplots of the pump pressures, intraluminal strain gauge pressures and pressure transducer values at injection velocities 4.5 and 8 mL/s, and the burst pressures of (b) the double lumen catheters; (c) the triple lumen catheters; and (d) the pre-curved catheters. *n* = 4 applies to (b–d). *Only three samples were tested.

All CVCs were successfully tested and calibrated by fitting a curve to the pressure and strain data. These curves were used to determine the intraluminal pressure from the strain gauge values during CM injection. As an example, Figure 3(a) shows the calibration data, curve fit and 95% confidence bounds of this fit in of a double lumen CVC tested. Figures S1 to S3 show the raw strain gauge data. The complete set of calibration figures can be found in Figures S4 to S6.

### Power injection of contrast medium

Figures 3(b) to (d) display the pump pressures, intraluminal strain gauge pressures (determined with the fitted calibration curves), pressure transducer values at injection velocities 4.5 and 8 mL/s, and the lowest recorded burst pressures of the three different CVC types. Pressure transducer measurements were lower than intraluminal pressures measured with the strain gauges in all instances. The intraluminal pressures were always lower than the pressure exerted by the pump. Intraluminal pressures always remained below the lowest burst pressure measured.

### Material fatigue

**Figure 4.**
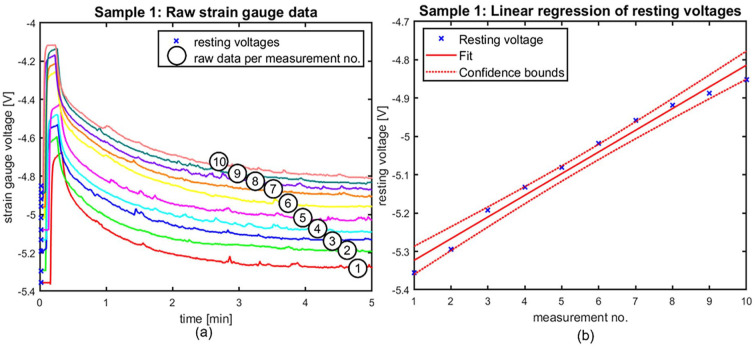
Representative regression plot of catheter 1 showing the trend in resting voltages throughout the measurements. Patterns are similar throughout the tested catheters. (a) shows the raw strain gauge data over the 5-min testing period of measurements 1 through 10 in catheter 1, as indicated by the numbers in the graph; and (b) displays the regression analysis of the resting voltages as indicated in (a).

Material fatigue, or permanent strain of the CVC, is assessed for each sample tested. Linear regression is applied to the strain gauge voltage at rest, which corresponds to the material strain at rest and fatigue. A non-zero regression coefficient thus corresponds to permanent strain of the material. The linear regression coefficients are non-zero (*p* < 0.01) in every CVC tested, except sample 2 (double lumen, *p* = 0.53). As an example, Figure 4 displays the raw strain gauge data of the first CVC measured together with the linear regression analysis performed on the resting voltages of this CVC. Figure S7 displays the linear regression analysis of CVC sample 2. The complete set of linear regression analyses is found in Figures S7 to S9.

### Burst pressure

**Figure 5.**
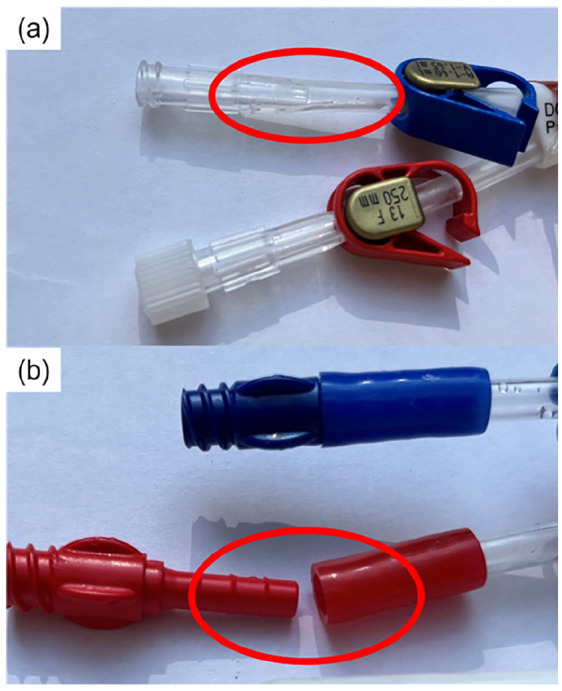
Failure modes of the central venous catheters at burst pressures: (a) failure of the inlet tube resulting in rupture and (b) dislocation of the inlet tube connector.

All CVCs failed at one of the locations displayed in Figure 5 during burst pressure measurement. Burst pressures and the failure location at burst pressure are also recorded in Table 1.

**Table 1.** Testing regime, burst pressure, and failure location for each catheter tested. Failure locations are shown in Figure 5.

Sample no.	CVC type	Testing regime	Burst pressure (kPa) ([PSI])	Failure location
1	Double Lumen	5 × 4.5 mL/s; 5 × 8 mL/s	1179 (171)	Inlet tube burst
2	Double Lumen	5 × 4.5 mL/s; 5 × 8 mL/s	N/A	N/A
3	Double Lumen	5 × 8 mL/s; 5 × 4.5 mL/s	1158 (168)	Inlet tube burst
4	Double Lumen	5 × 8 mL/s; 5 × 4.5 mL/s	1110 (161)	Inlet tube burst
5	Triple Lumen	5 × 4.5 mL/s; 5 × 8 mL/s	862 (125)	Inlet tube burst
6	Triple Lumen	5 × 4.5 mL/s; 5 × 8 mL/s	917 (133)	Inlet tube burst
7	Triple Lumen	5 × 8 mL/s; 5 × 4.5 mL/s	N/A	N/A
8	Triple Lumen	5 × 8 mL/s; 5 × 4.5 mL/s	800 (116)	Inlet tube burst
9	Pre-Curved	5 × 4.5 mL/s; 5 × 8 mL/s	1165 (169)	Connector dislocation
10	Pre-Curved	5 × 4.5 mL/s; 5 × 8 mL/s	1248 (181)	Connector dislocation
11	Pre-Curved	5 × 8 mL/s; 5 × 4.5 mL/s	1220 (177)	Connector dislocation
12	Pre-Curved	5 × 8 mL/s; 5 × 4.5 mL/s	N/A	N/A

### Microscopic surface analysis

**Figure 6.**
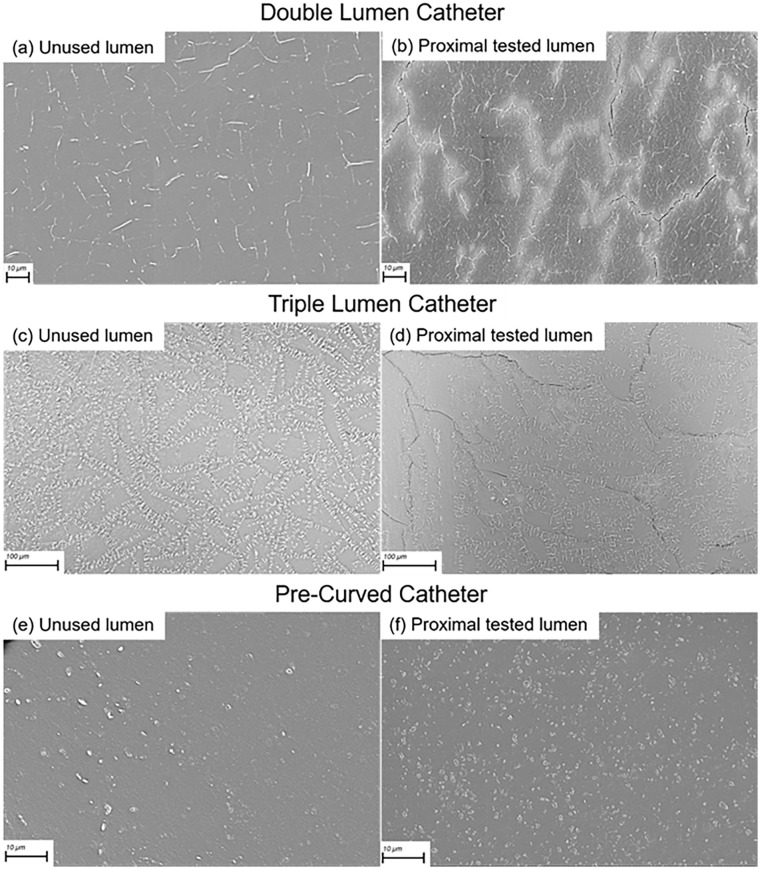
Surface analysis through scanning electron microscopy of: (a) unused lumen, and (b) part from the inlet side of the tested lumen, of the double lumen catheter; (c) unused lumen, and (d) part from the inlet side of the tested lumen of the triple lumen catheter; (e) unused lumen, and (f) part from the inlet side of the tested lumen, of the pre-curved catheter. Small white dots and lines visible in all images are identified as pores in the material. The black lines are indicative of micro-cracks. There are notable differences in the luminal surfaces across the various types of catheters. The magnifications used were selected to best illustrate the surface changes in each catheter type before and after exposure to contrast medium injection.

Magnification was chosen to best illustrate the differences in the surface in each CVC. The microscopic surface analysis of the three CVCs examined is depicted in Figure 6. With the unused lumina as control, the pictures of the tested lumina show an increase in size and density of micro-cracks in the double and triple lumen CVCs. These micro-cracks present themselves as clear black lines in the microscopy images. However, this contrast is less apparent in the pre-curved CVC, where the lumen was larger (15.5 F vs 13 F) and intraluminal pressures were lower.

## Discussion

In this study it was observed that intraluminal pressures during power injection of CM remain well below burst pressure. By using strain gauges and a pressure transducer in the flow circuit, the data suggest that strain gauges provide a more accurate measurement of intraluminal pressure. Moreover, the strain gauges showed that material fatigue and damage can occur to the CVCs with repeated use. Surface analysis of the CVCs was performed to assess microscopic damage to the material, in which a greater incidence of micro-cracks was noticed after testing. This data can guide clinicians in responsible use of CVCs for CM power injection.

As in literature,^6,8–12^ the measured pressures were well below bursting pressure. Bursting pressures were also comparable.^6,10^ The CM injector pressure was higher than the intraluminal pressure, which is necessary to overcome the frictional losses in the tubing and CVCs. Given that the injection pressures through CVCs are comparable to those in standard angiography catheters^21^ and are applied for only a short duration, the risk of vascular or cardiac tissue damage is expected to be minimal.

The outliers in the injector pressure may be explained by the injector exerting an increased pressure on the fluid bag when almost empty, which was a constant trend. The intraluminal pressures also show a substantial variation in values, explained by the sensitivity of the gauge to environmental factors and the measurement error in the calibration.

Contrarily to the injector pressures, the intraluminal pressures in the different types of CVCs show some discrepancy, likely because the lumina have dissimilar cross-sections. The highest intraluminal pressures correspond to the smallest cross-sectional area. Despite the geometrical differences between the lumina of the pre-curved CVCs, the injector pressures were similar through both when injecting at the same velocity. This suggests pressures are also similar in both lumina.

Although the CM injector exerted greater pressures at higher injection velocities, the intraluminal pressures appear more similar. An explanation could lie in the frictional losses of the fluid flowing through a substantial length of tubing before entering the CVC. Frictional losses are greater at higher velocities and small diameters, which explains considerable pressure losses. Additionally, the three-way stopcock present in the flow circuit may have introduced more turbulence in the flow which could have resulted in a slight drop in pressure and affected the measurements downstream.

The pressure transducer placed further upstream should measure a higher pressure than the strain gauges. Therefore, it is an interesting finding that the pressure measured by the pressure transducer was noticeably lower than the pressures measured by the strain gauges. However, a three-way connection is placed to connect the pressure transducer which introduces dead space in which fluid is static. This likely interferes with the flow, as these stagnant zones act as reservoirs of pressure, exerting localized effects that can distort the overall pressure profile in such locations. Turbulent eddies may form in regions of abrupt flow constriction or expansion, causing fluctuations in pressure and velocity. In systems with rapidly changing flow rates, the presence of dead space can thus impede the propagation of pressure waves and dampen fluid oscillations, leading to distorted pressure transducer readings.^20^ This dead space will thus not always show a direct pressure response to fluidic flow, which impedes accuracy of the measurement. These phenomena may contribute to the rather large differences in measured pressures throughout previous in vitro studies (2–70 PSI at 4.5 mL/s velocities with similar catheters).^6,7,11,12^ As the strain gauges do not interfere with the flow and they measure pressure directly at the location of interest, the pressures they measured are more likely to be valid.

The fatigue analysis of the strain values implies that material damage accumulates throughout the repeated measurements. Consistency in peak amplitudes suggests that the adhesive interface between the strain gauge and CVC remained intact. In all CVCs, except one, this coefficient is statistically significant. In CVC sample 2 the non-significant coefficient appears to result from the last two measurements having a far higher resting voltage. This likely occurred through accidental interference with the CVC during testing, and registered by the high sensitivity of strain gauges. It is likely that higher CM volumes or flow rates will accelerate the fatigue process.

The microscopy shows an increase in size and density of micro-cracks between the unused and tested luminal surfaces in the double and triple lumen CVCs. These cracks seem to propagate along what appear to be small pores in the material. Propagation is likely induced by the pressure of the CM injection. Electron microscopy in literature of similar dialysis CVCs, exposed to normal use and explanted from patients, do not show such clear micro-cracks,^22,23^ suggesting the CM injection caused the damage. These micro-cracks could potentially promote bacterial adhesion and biofilm formation. This study does not address such risks, but proper handling and flushing remains crucial.

In the pre-curved CVCs, no such micro-crack formation is clearly visible. This could be due to the intraluminal pressures in the pre-curved CVCs being lower due to the larger cross-sectional area of these lumina, thereby causing less damage.

As the CVC material appears to stretch after CM injection, permanent stretching and offset may have occurred prior to calibration. The validity of calibration data can thus be questioned. Although most regression coefficients are significantly non-zero, the actual changes in resting voltage are relatively small compared to the amplitude of the voltage peaks. Moreover, the amplitudes of the voltage peaks remain fairly constant throughout the five measurements at the different injection velocities in all CVCs. The effects on the determined intraluminal pressure values are therefore expected to be limited.

Failure of the CVCs at bursting pressure always occurred at the inlet, which in clinical use will remain outside the body. However, the stretching of the material and increase in micro-crack size and density suggested damage accumulates with repeated CM injection. The micro-cracks may increase thrombus risks but this remains to be studied. With the velocities tested, incidental use likely carries little risk.

The relatively small number of CVCs used does pose issues with drawing statistically significant conclusions, and statistical analysis has therefore been omitted. However, the burst pressures measured were always at least a factor 2 higher than the intraluminal pressures measured which does illustrate a substantial margin of safety. Burst pressure measurement was only performed on three CVCs of each type, but they do resemble values previously found in literature.^6,10^ The electron microscopy on single samples holds no statistical value, but does support the findings on pressure and fatigue in all the CVC types tested, and aids in understanding effects of repeated use. Future studies should include larger sample sizes.

This study has several limitations. Intraluminal pressures were measured in vitro, not fully simulating in vivo conditions, and the CVCs are not subjected to normal clinical handling which may impact device integrity. Although CVC pressures during dialysis typically do not exceed arterial pressure, the CVCs were new and not subjected to normal dialysis use. They were also not maintained at body temperature. Testing at body temperature could have increased flexibility of the material. Contrarily, rigidity, and thus pressure, could increase due to fibrous tissue forming on the CVC in the body. Venous pressure at the tip was absent, which may have increased intraluminal pressure. However, venous pressure is typically far lower than the pressures measured in the system. Moreover, intraluminal pressure is only measured at one location in the catheters. Although pressure close to the inlet is expected to be highest, the pressure and stress profile throughout the entire CVC is not examined. Therefore stress or pressure concentrations that could occur are not considered. Additionally, a lower temperature than typically used in patients was used with a high-concentration CM (350 mg/mL iodine) which will have considerably increased the viscosity^21^ and resulting pressures will therefore have been overestimated. It has been found that higher concentrations up to the typical maximum of 400 mg/mL may further increase pressures by ~15%.^21^ Any changes in fluid composition due to evaporation or deposit on the luminal surface during reuse of the CM are not considered. However, this will likely only have increased the concentration of the CM, and thus further overestimated in-use pressures. Moreover, the fatigue analysis suggests that stretching of and damage to the material occurs after repeated use. However, it is not known how material fatigue relates to material strength, and to which extent safe use may be maintained. In vivo factors and fatigue should receive attention in future safety studies.

Finally, CVCs are medical devices regulated under the European Medical Device Regulation in the EU^24^ and the FDA in the US.^25^ Manufacturers must meet performance and safety requirements for market approval,^24^ based on the device’s intended use. Expanding use cases thus requires additional evidence, leading manufacturers to limit intended uses to reduce costs. Despite reports of safe in vitro use with higher velocities^6–12^ and clinical application,^13–15^ incidents of rupture have been reported.^16–18^ Unexpected tip displacement was found to increase thrombus risk, which may be addressed with adequate flushing.^18^ Guidelines for CM injection through CVCs are limited^19^ and most standard CVCs remain uncertified for CM injection. This likely causes hesitance in their use for such procedures, in which liability is shifted to the healthcare professionals. Clinical use of CVCs for this purpose has previously been shown to be safe with sufficient image quality, provided adequate protocols with pressure limits are in place.^14^ However, caution must be taken and caretakers must understand that liability is shifted away from the manufacturer when using such CVCs for CM injection.

The findings presented highlight that while incidental use of CVCs for CM injection does not pose immediate risks, repeated use could compromise catheter integrity over time. This research underscores the importance of cautious, informed application of CVCs for CM injections in clinical settings, guiding practitioners toward safer, evidence-based decisions to prevent potential complications.

## Supplemental Material

sj-pdf-1-jva-10.1177_11297298251333014 – Supplemental material for In vitro safety of power injection of contrast media through central venous hemodialysis catheters Supplemental material, sj-pdf-1-jva-10.1177_11297298251333014 for In vitro safety of power injection of contrast media through central venous hemodialysis catheters by Nicholas A White, Aart J van der Molen, Ronald W A L Limpens, Jacinta J Maas, Koen E A van der Bogt, Tim Horeman and Joris I Rotmans in The Journal of Vascular Access
