# Supplementary material for: In vitro safety of power injection of contrast media through central venous hemodialysis catheters
Source: J Vasc Access. 2025 Apr 14;27(1):190–200. doi: 10.1177/11297298251333014 (PMC12812178; doi:10.1177/11297298251333014)
Supplement: sj-pdf-1-jva-10.1177_11297298251333014 – Supplemental material for In vitro safety of power injection of contrast media through central venous hemodialysis catheters [file sj-pdf-1-jva-10.1177_11297298251333014.pdf]

Intraluminal pressure in central venous hemodialysis catheters during power injection of contrast media

Supplemental File 1: Strain Gauge Calibration and Fatigue Analysis

Supplemental Figures S1-S9

Raw data

Double Lumen Central Venous Catheters

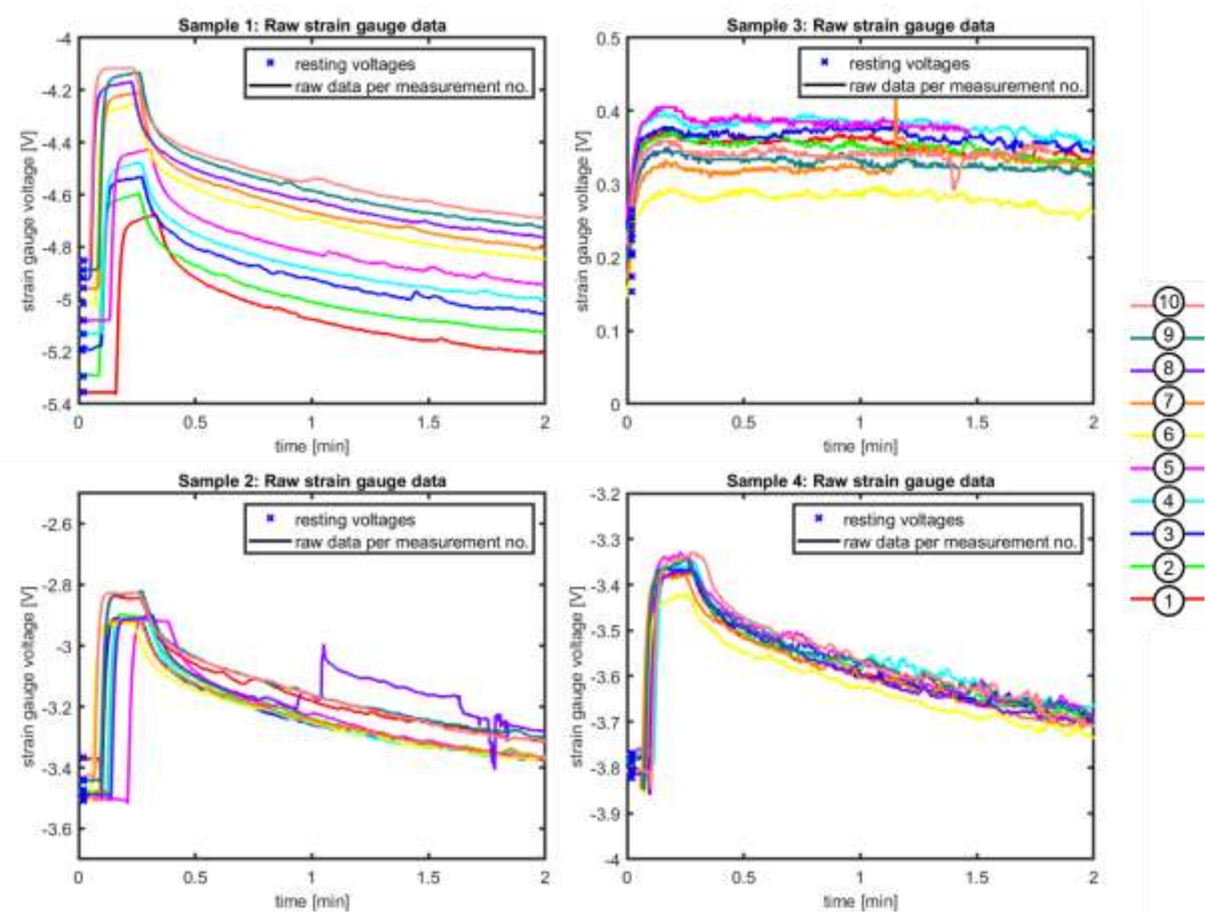

Figure S1: Raw strain gauge of the Double Lumen Central Venous Catheter samples. Only the first 2 minutes of the 5-minute measurement intervals are shown to more clearly depict the strain gauge response to changes in flow and pressure. The measurement number corresponding to the line number can be found on the right of the graphs. Note: the measurement distortion sometimes seen in the right parts of the measurement illustrate the sensitivity of sensors to movement, and are usually caused by accidental movement of the test setup. These values have been filtered out from the processed data.

Triple Lumen Central Venous Catheters

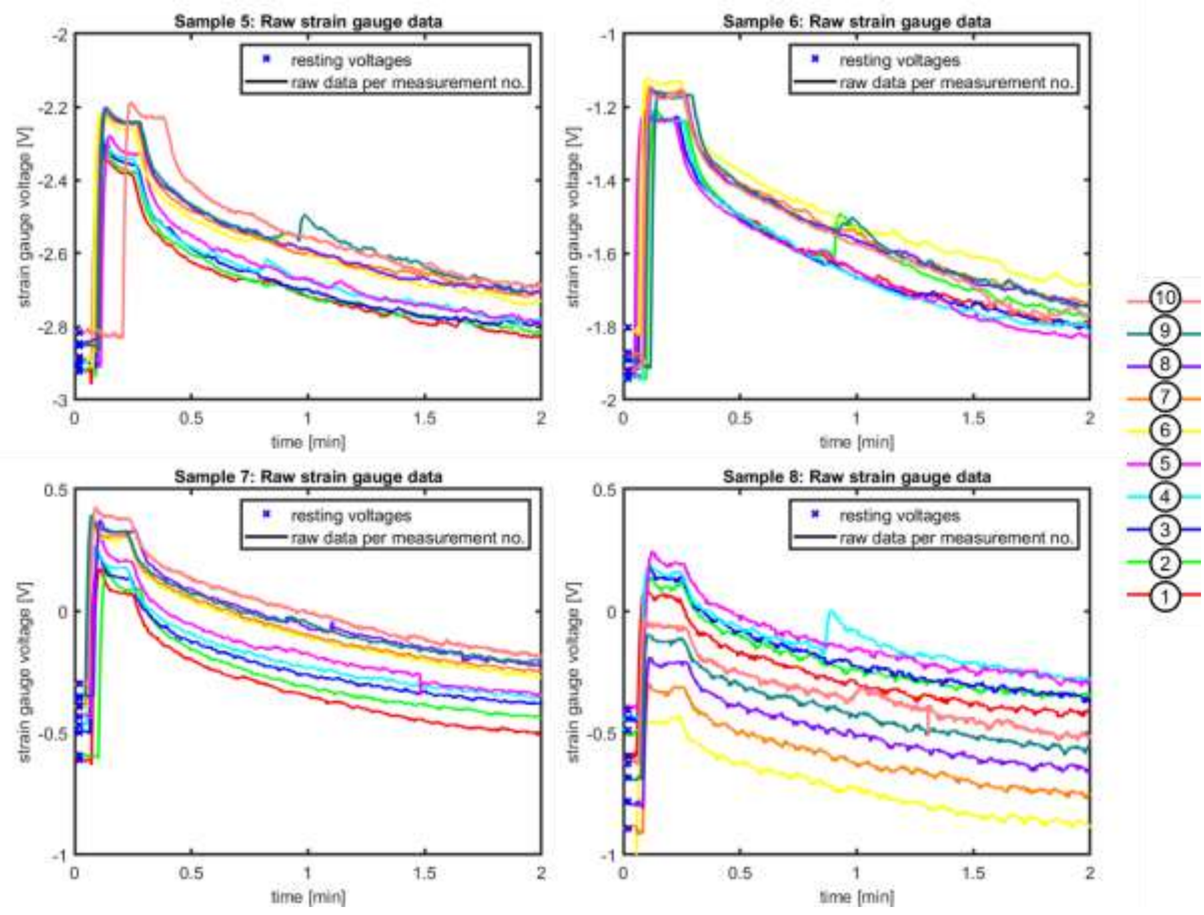

Figure S2: Raw strain gauge of the Triple Lumen Central Venous Catheter samples. Only the first 2 minutes of the 5-minute measurement intervals are shown to more clearly depict the strain gauge response to changes in flow and pressure. The measurement number corresponding to the line number can be found on the right of the graphs. Note: the measurement distortion sometimes seen in the right parts of the measurement illustrate the sensitivity of sensors to movement, and are usually caused by accidental movement of the test setup. These values have been filtered out from the processed data.

Pre-Curved Central Venous Catheters

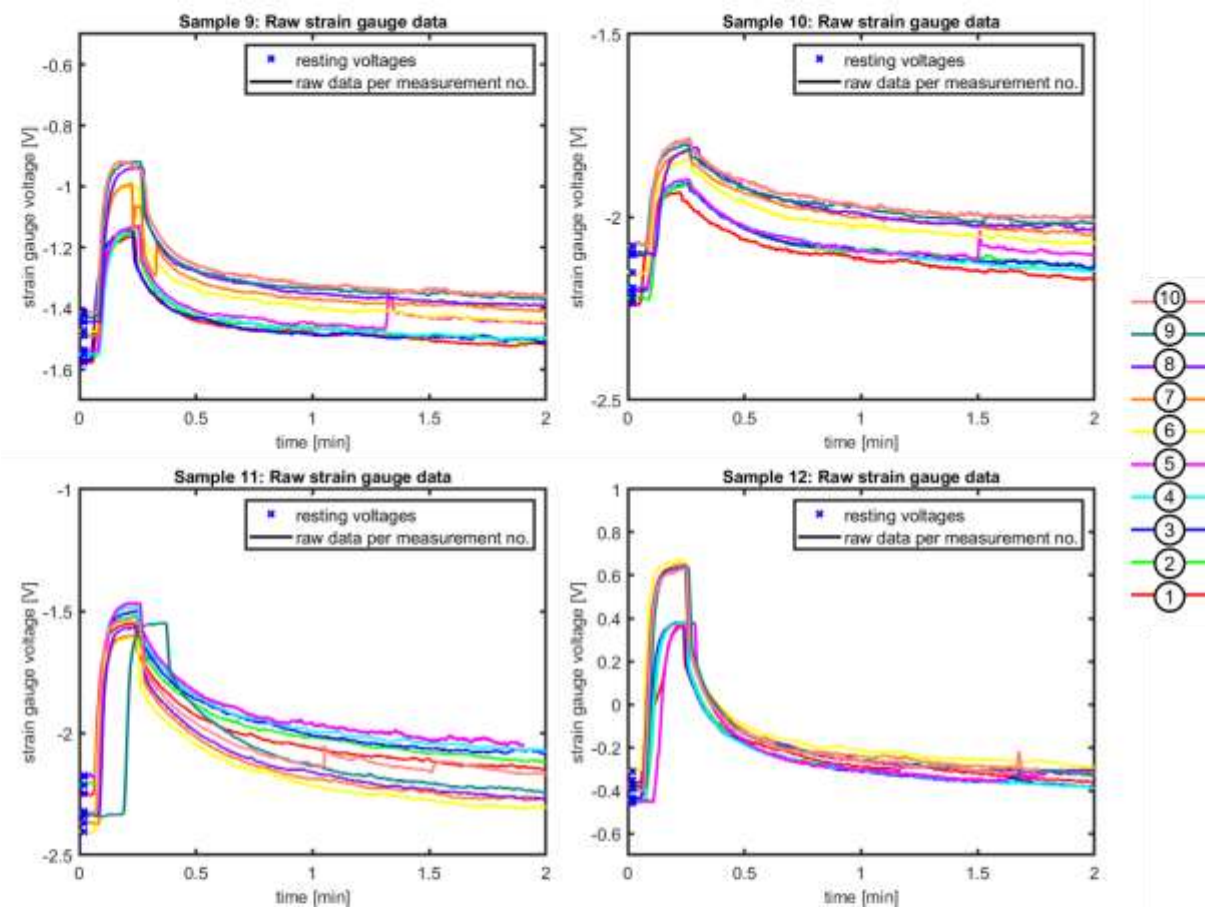

Figure S3: Raw strain gauge of the Pre-Curved Central Venous Catheter samples. Only the first 2 minutes of the 5-minute measurement intervals are shown to more clearly depict the strain gauge response to changes in flow and pressure. The measurement number corresponding to the line number can be found on the right of the graphs. Note: the measurement distortion sometimes seen in the right parts of the measurement illustrate the sensitivity of sensors to movement, and are usually caused by accidental movement of the test setup. These values have been filtered out from the processed data.

Strain Gauge Calibration

Double Lumen Central Venous Catheters

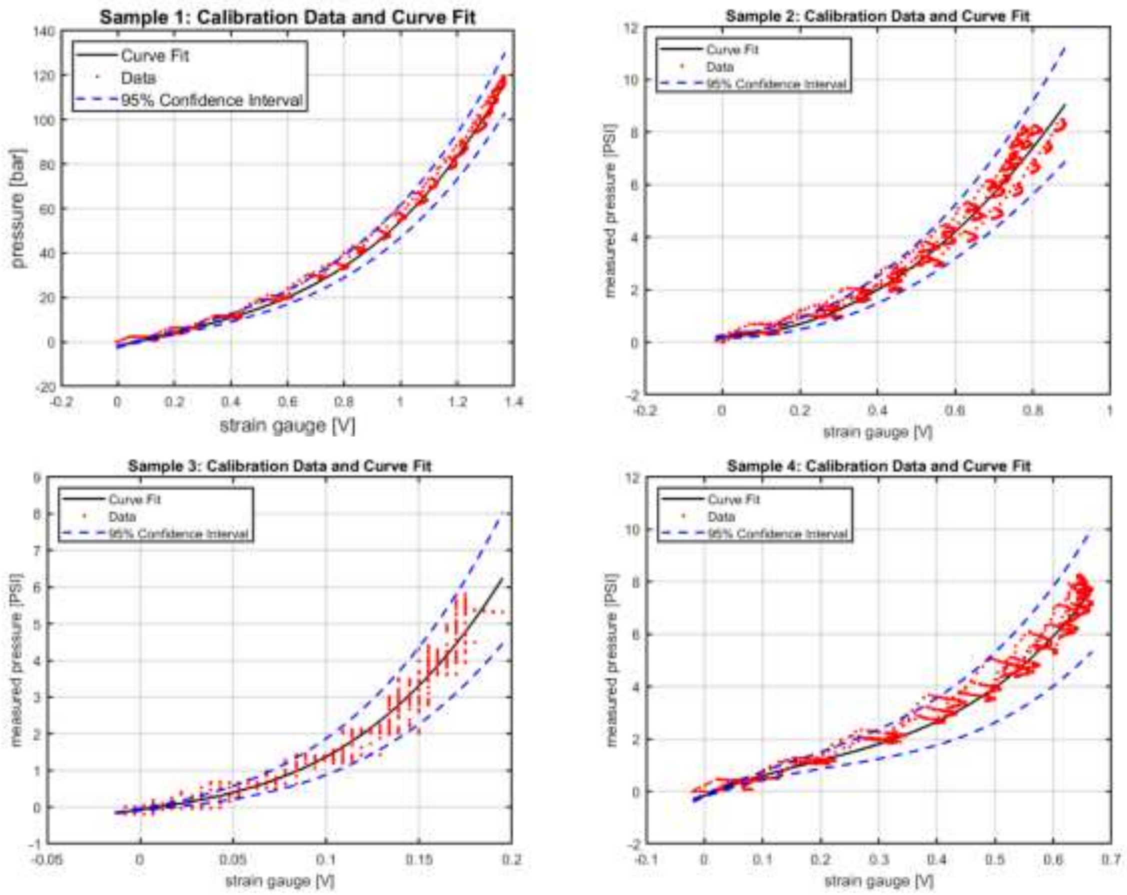

Figure S4: The calibration data, fitted 3rd-order polynomial and its 95% confidence bounds of catheter sample 1-4 (double lumen), used to determine intraluminal pressure with the strain gauge.

Triple Lumen Central Venous Catheters

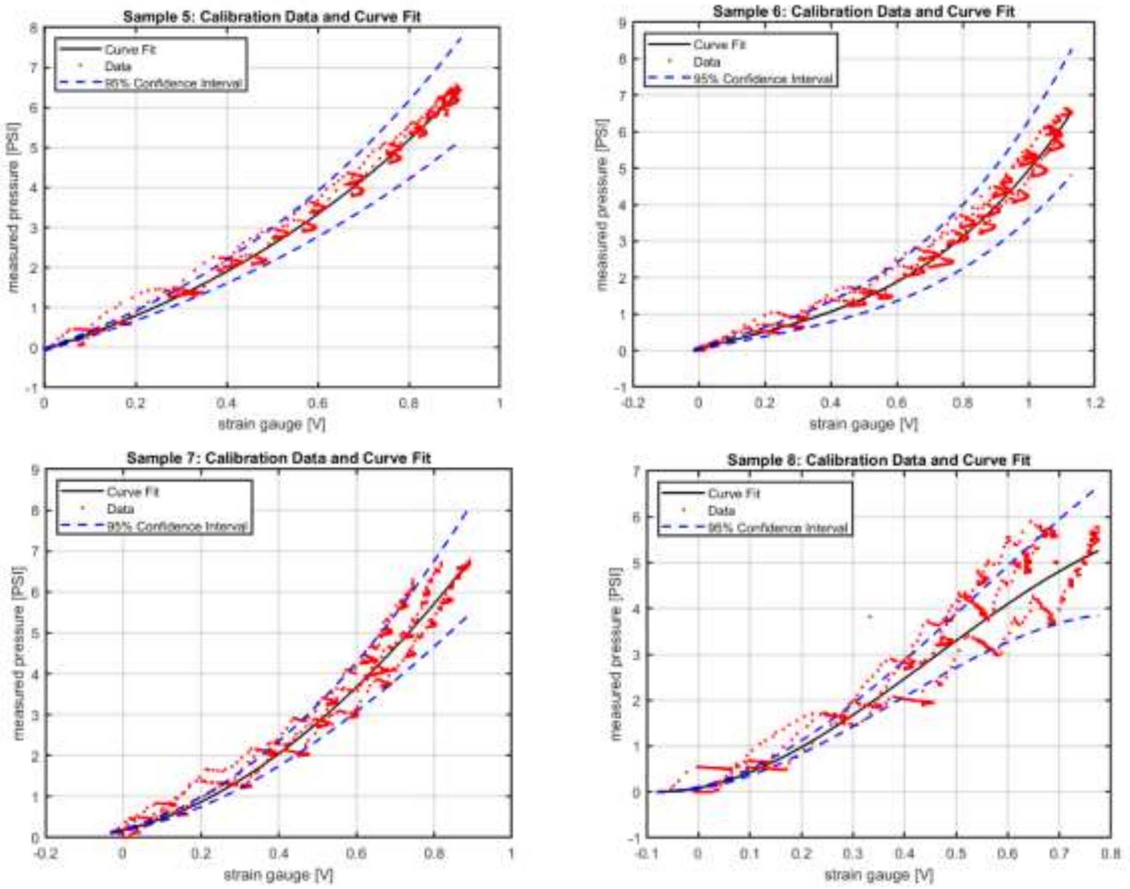

Figure S5: The calibration data, fitted 3rd-order polynomial and its 95% confidence bounds of catheter samples 5-8 (triple lumen), used to determine intraluminal pressure with the strain gauge.

Pre-Curved Central Venous Catheters

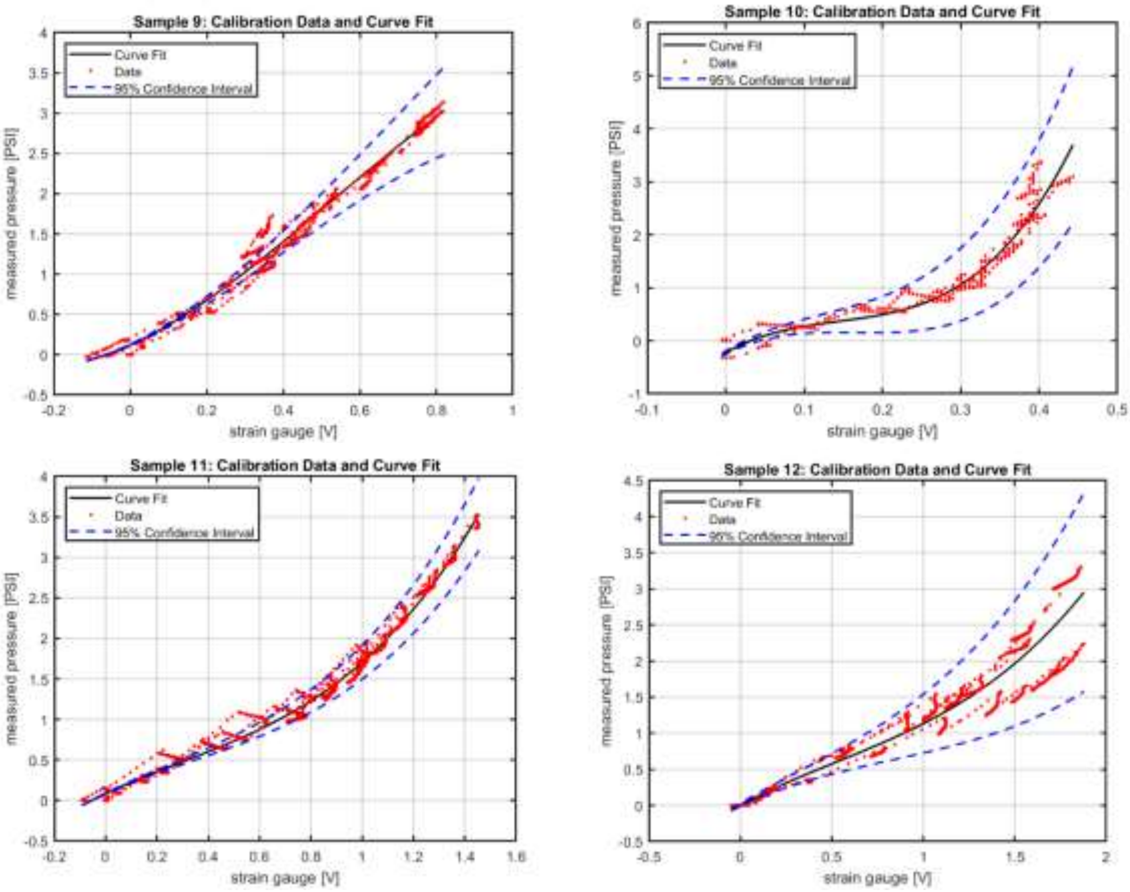

Figure S6: The calibration data, fitted 3rd-order polynomial and its 95% confidence bounds of catheter samples 9-12 (pre-curved), used to determine intraluminal pressure with the strain gauge.

Material Fatigue Analysis

Double Lumen Central Venous Catheters

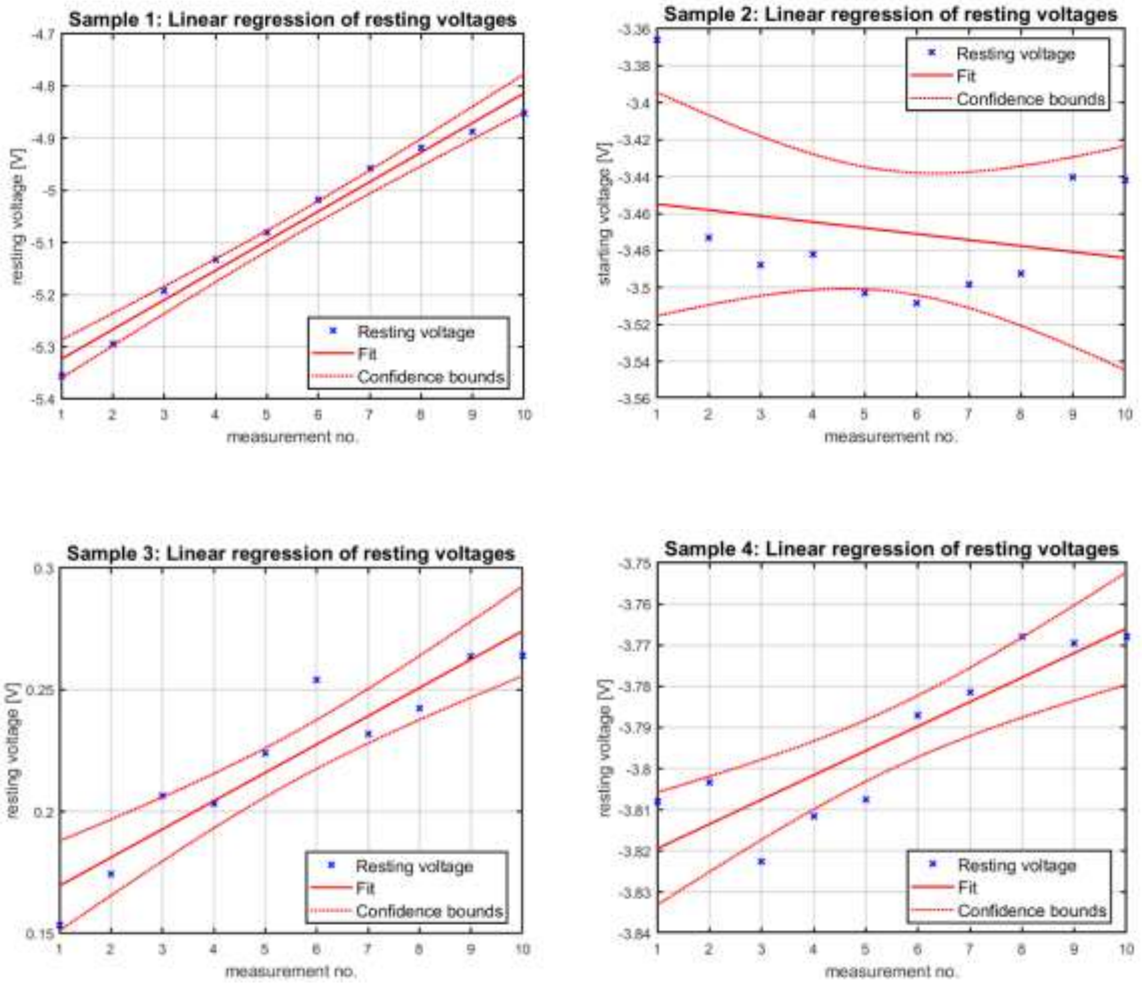

Figure S7: Linear regression analysis, together with 95% confidence bounds, on resting voltages versus measurement numbers to determine material fatigue on catheter samples 1-4 (double lumen).

Triple Lumen Central Venous Catheters

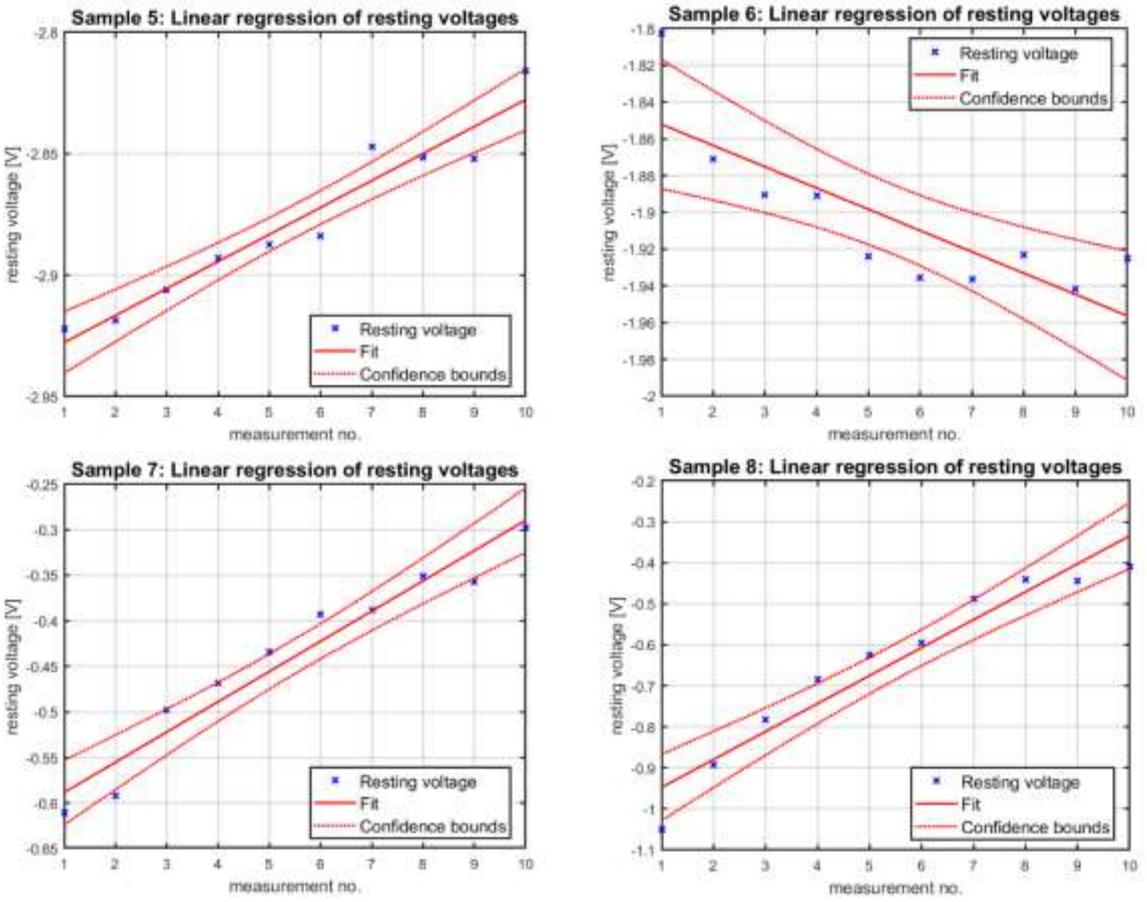

Figure S8: Linear regression analysis, together with 95% confidence bounds, on resting voltages versus measurement numbers to determine material fatigue on catheter samples 5-8 (triple lumen).

Pre-Curved Central Venous Catheters

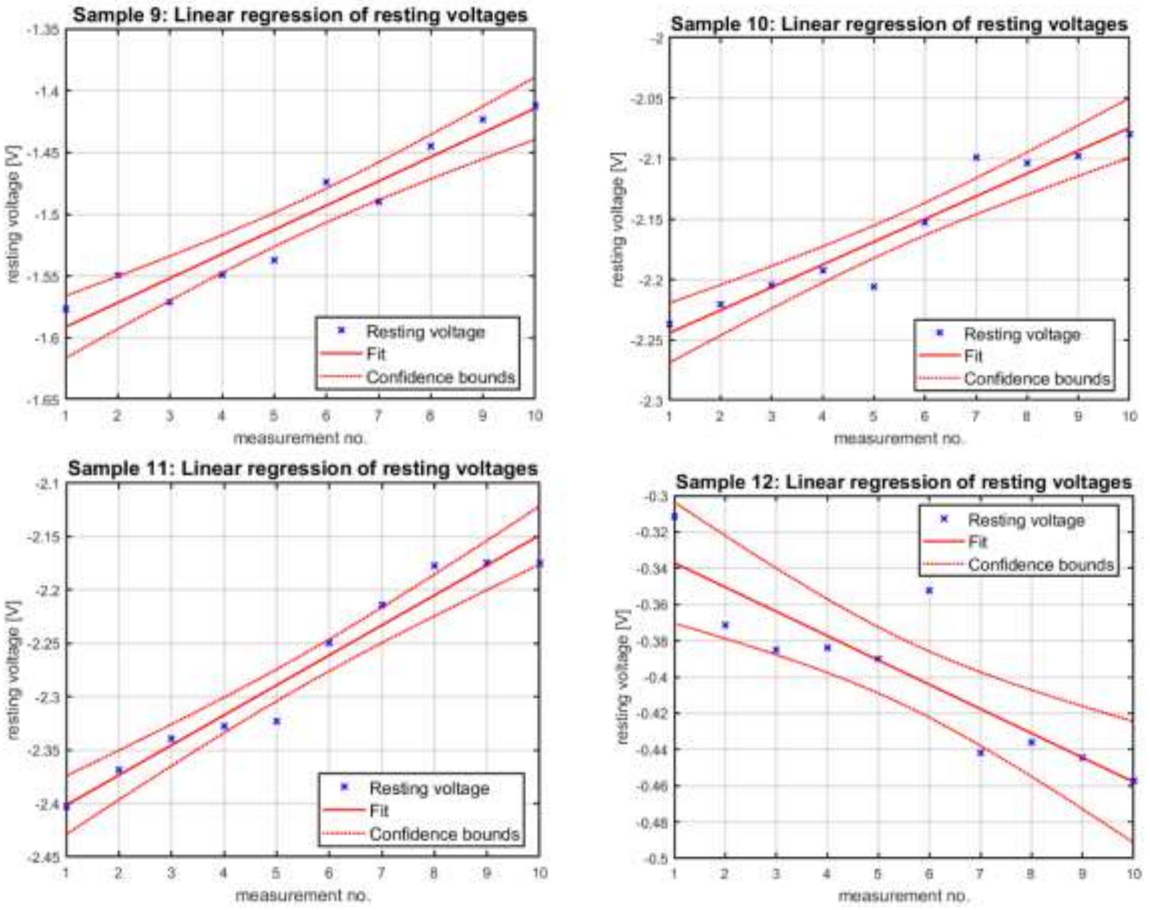

Figure S9: Linear regression analysis, together with 95% confidence bounds, on resting voltages versus measurement numbers to determine material fatigue on catheter samples 1-4 (double lumen).
